# Alveolar Abscess

**Published:** 1868-09

**Authors:** W. H. Shadoan


### ALVEOLAR ABSCESS.

BY DR. W. H. SHADOAN.

[Continued from page 346.]

### TREATMENT.

By treatment is meant all means employed for the cure of disease. The treatment of abscess may be divided into three classes, *Preventive*, *Therapeutic*, and *Surgical.* The preventive may embrace all means employed, and in order to be the better understood and to avoid confusion, we will arrange our classes of treatment, commencing with

### PREVENTIVE.

In this class of treatment we often have to resort to general as well as local means. An abscess may, in some instances, be prevented by antiphlogistic treatment, such as saline cathartics, leeching the gums, and anything of a cooling nature, either constitutionally or locally applied. There are so many different remedies for different cases that it is impossible to give a single remedy that will suit all. Where we wish to prevent the formation of an abscess the first and most important thing to be done is to remove the exciting cause. If the pulp be dead in a tooth, its removal will, in many cases, prevent the formation of an abscess or the removal of any foreign body, such, for instance, as the roots of dead teeth, or any thing that proves an exciting cause. Sometimes the application of a counter-irritant to the gums, when the use of leeches is impracticable. The gums may be scarified and bathed in warm water to promote bleeding which will, usually, prove beneficial. Painting the gums well with compound tinct. of iodine is also attended with good results. I have also found great relief in the application of croton oil to the gums; a saturated solution of creosote and iodine as a local application is invaluable in many cases. For the last year or two I have been using, with great satisfaction, "*Mercurius vivus*," (third trituration,) which, for acute periosteal inflammation, in many cases has no rival, except where the system is very susceptible to impressions of mercurial agents, then it should not be used. If the case is found to be too obstinate to yield to the preventives used, the only course left for us is to use palliatives until the sac is formed and then adopt the kind of treatment indicated.

"Pressure will limit the outward wave whenever applied upon a purely healthy portion of structure, and define the size of an abscess by turning back the wave of nutrient activity in the deteriorated part upon itself, preserving the outward sea from threatened disturbance." Vigorous or active exercise, both bodily and mental, have a powerful influence on incipient abscess, and sometimes cure entirely. Why, or how this is done may not be very well understood, nevertheless it is true, and may be accounted for by the inviting away of the nerve force and current of blood and literally starving the abscess. When therapeutic agents are used the principle of their action is very much the same as that of pressure in arresting the wave of poisonous nutrition, and in like manner they abate or greatly reduce the size of the sac.

### THERAPEUTIC TREATMENT.

Of the remedial agents for the cure of alveolar abscess we will mention *creosote*, *nitrate of silver*, *chloride of zinc*, *chlorate of potassa*, *iodine*, *iodide of potassium*, *bromine*, and *bromide of potassium*, etc.

### CREOSOTE.

*Creosote* was discovered by Reichenbach, of Blenkso, in 1830; it is procured by the dry distillation of various vegetable as well as animal substances, but is officinally described to be obtained from wood tar. It is a colorless, oily liquid, of a peculiar, disagreeable and penetrating odor, resembling that of wood smoke, and has a burning, acrid taste which is perceived throughout the whole extent of the buccal, nasal, and pharyngeal mucous membrane. Its specific gravity at 68° F. is 1.037. It boils at 397° F., and is frozen at 17° F. It burns with difficulty in the air, emitting large volumes of smoke. It coagulates albumen but exerts no action upon fibrin. Owing to these facts, and its strong antiseptic properties, it is considered one of the best therapeutic agents in the cure of all ulcerative affections. This substance is more peculiarly adapted to the treatment of alveolar abscess than any other known agent. "Creosote is an active caustic. Its escharotic power is due to its affinity for albumen, which is so strong as to take the latter from living tissue and thus destroy vitality to the extent of the combination. Its compcund with albumen is white, hence the extent of its escharotic action is readily observed." In addition to its strong affinity for albumen with which it rapidly forms a permanent insoluble compound, it possesses the valuable property of arresting and preventing the decomposition of animal matter, which renders it preferable to any other agent that has hitherto been introduced for the treatment of abscess. "Its great penetrating power enables it to pervade every part of the cavity and diffuse itself over the entire surface of the sac, thus effectually securing the desired result." It is also one of the most energetic stimulants.

The mode of applying *creosote* is usually by injection. In some cases, however, where the fistulous opening is large it may be applied by saturating a pledget of cotton or lint and forcing it into the cavity formed by the abscess. The most successful means of applying creosote is by injection. I find this most readily done by the use of a syringe when the liquid is to be inserted through the fistulous opening, but if the root of the tooth is well opened then the pulp cavity may be filled with gutta percha or Hill's stopping, through which drill a hole for the insertion of the point of the syringe which will greatly facilitate the operation. Another manner of injecting an abscess is preferable to the above, inasmuch as it is less complicated and will allow a much more thorough operation than with the syringe especially if the foramen be small. This is accomplished by shaping a broach of a piece of pivot or any hard wood, such as straight grained hickory, barb the point, around which roll loosely some cotton, this dip into the creosote and use as a piston, dipping it into the creosote every few seconds and forcing it into the sac until the patient complains of pain. If there be a fistulous opening through the gums the creosote can, in nearly all cases, be forced through the tooth, sac, and gum. This is indicated by the surface around the fistulous opening turning white, and is an indication that the process has been carried far enough for that time. If the operation has been thoroughly performed, the creosote having pervaded every particle of the sac, there is little doubt that a cure will be effected, other things being favorable. I find, in many cases, a cure may be effected in healthy patients with a single application of the creosote. In most cases all can be accomplished with creosote that can be with other agents, I seldom use anything else for that purpose. Were it not necessary in a paper like this to give all the remedies used I would let the above suffice, but to meet the views of all, other remedies will be given.

### NITRATE OF SILVER.

Although this remedy is but little used, it is occasionally employed and is deserving a place in the catalogue of *remedies*, a consideration of its merits will not be regarded out of place.

*Nitrate of silver* is a white salt having an intensely metallic, bitter taste, it is usually prepared in the form of hard, brittle sticks, at first it is white but becomes gray, and afterward more or less dark under the influence of the air. It was once thought that the light caused this substance to turn dark, but this is shown to be erroneous. The turning dark is caused by organic matter or sulphuretted hydrogen contained in the air. Silver coin will be affected in the same way. Its affinity for animal matter is evinced by its forming definite compounds with albumen and fibrin. *Nitrate of silver* is soluble in its own weight of water and in four parts of alcohol, its solution stains the skin an indelible black color; when exposed to heat it fuses at 426°, and at 600° undergoes decomposition with evolutions of oxygen and nitrous acid.

*Nitrate of silver* is employed as a vesicant, stimulant, and escharotic, either dissolved in water or in the solid state. It may be used in abscess for two purposes, one to break up the sac, the other to heal the ulcer, and when employed for the latter should be applied in solution of about two or three grains to the fluid ounce of water; a drachm of the salts to an ounce of water forms an escharotic, and may be injected into the sac of an abscess. I usually prefer the solid nitrate to the solution. I find the best and most convenient mode of applying the nitrate in the solid state is by pulverizing the crystals as finely as possible, and by means of a small slightly tapering silver tube they may be carried into the sac with little difficulty. There is another way of introducing the nitrate that may be well to mention; take a small stick of the salts, about the size of the lead in a common pencil, and upon it form a covering of melted engraver's wax, this done, trim off a portion of the wax at the point and insert it rapidly, not allowing time for the substance to dissolve and stick to the flesh. This method is not so successful or satisfactory as the other. The injecting of the solution may be conducted in the same way as that of creosote.

### CHLORIDE OF ZINC.

As this remedy is seldom used I shall not enter into a detailed account of its administration and uses. The chief employment of the chloride of zinc has been externally as an escharotic, applied to schirrhus and cancerous affections and to ulcers of an anomalous and intractable character. When thus used it acts not only by destroying the diseased structure but by exciting a new and healthy action in the surrounding parts. It may be applied in the same manner as nitrate of silver. The greatest benefit resulting from the use of chloride of zinc is in the reproduction of alveolar process, where it has no rival. The chloride should not be used oftener than once a day for one or two days. Then, if the application has been thorough, there is no further use for this agent.

TO BE CONTINUED.

